# Recovery from Borderline Personality Disorder through Dialectical Behavior Therapy

**DOI:** 10.46743/2160-3715/2017.3008

**Published:** 2017-11-26

**Authors:** Carla D. Chugani, Ashley R. Seiler, Tina R. Goldstein

**Affiliations:** University of Pittsburgh, Pittsburgh, Pennsylvania, USA

**Keywords:** Borderline Personality Disorder, Recovery, Dialectical Behavior Therapy, Thematic Analysis

## Abstract

This article presents a qualitative investigation of the perspectives and experiences of recovery from borderline personality disorder from six individuals who were treated with comprehensive dialectical behavior therapy. Data were collected via semi-structured interviews, transcribed, and coded using a six-step analysis process. Six primary themes emerged: (1) belief about recovery, (2) current experience of self, (3) facets of recovery, (4) motivating factors, (5) external supports to recovery, and (6) characteristics required for recovery. Overall, the findings took a dialectical form in which participants often described conflicting experiences (e.g., feeling recovered while also continuing to experience heightened emotional sensitivity). We conclude that the themes presented in this article represent broad domains related to the meaning of recovery from BPD, and recognize that the relative importance of each domain is best determined by the individual.

## Introduction

Dialectical Behavior Therapy (DBT) was originally developed to treat individuals with high-risk suicidal and self-injurious behavior (Linehan, 1993). Since its inception, DBT has been established as a best practice for the treatment of borderline personality disorder (BPD) through numerous randomized controlled trials (e.g., Koons et al., 2001; Linehan, Armstrong, Suarez, Allmon, & Heard, 1991; Linehan et al., 2006). DBT has been listed in the National Registry of Evidence-Based Practices for the last decade (Substance Abuse and Mental Health Services Administration, 2006) and is considered to be the most intensely studied treatment for BPD (Stoffers et al., 2012). The overarching goal of DBT is to help clients develop lives worth living (i.e., developing a life that the individual feels is fulfilling, satisfying, and “worth living” such that suicide is no longer desirable). In contrast, research related to DBT has largely focused on the outcomes that are most logically connected to its efficacy in reducing BPD symptoms and suicidal behavior (i.e., the literature has focused primarily on symptom remission as the primary outcome). While this type of outcome measurement is both logical and necessary in demonstrating the efficacy of any intervention, we assert that symptom remission and recovery are interrelated but distinct concepts, both of which merit investigation. The purpose of this study was to examine the lived experiences of individuals who have recovered from BPD through treatment with DBT in hopes of developing a richly detailed understanding of what it means to be “recovered” from the patient perspective.

## Literature Review

### Clinical Perspectives

A small body of literature attempts to examine the course of remission and recovery from BPD. A particularly rigorous, longitudinal study of remission and recovery from several mental health disorders, including BPD, is the McLean Study of Adult Development (for an overview, see Zanarini, Frankenburg, Hennen, Reich, & Silk, 2005). Zanarini and colleagues (2012) define remission from BPD as no longer meeting the diagnostic criteria for the disorder for a period of two years or longer. Those authors conceptualize recovery from BPD as more complex, including symptom remission, a global assessment of functioning (GAF) score of at least 61, and, “at least one emotionally sustaining relationship with a close friend or life partner/spouse, and be able to work (including work as a houseperson) or go to school consistently, competently, and on a full-time basis” (Zanarini et al., 2012, p. 2). For individuals with BPD over a 16-year period, remission was more common than recovery, and sustaining either remission or recovery was more challenging for individuals with BPD as compared to individuals with another personality disorder (Zanarini et al., 2012).

Furthering this work, Ng, Bourke, and Grenyer (2016) conducted a systematic review of the literature related to recovery from BPD. While individuals with BPD demonstrated substantial improvements in functioning across studies, they generally continued to struggle with various difficulties that impaired functioning. Further, individuals with BPD are likely to have longer times to remission relative to individuals with other personality disorders or major depressive disorder, but not schizophrenia. Although the majority of the articles reviewed by these authors were quantitative in nature, they also synthesized the results of three qualitative studies. Across these studies, three main themes emerged: (1) Active willingness to engage in recovery journey, (2) Improving on clinical characteristics of BPD to facilitate change, and (3) The conceptualization of recovery (i.e., whether or not the word “recovery” defined their experiences, or alternative views of recovery as a process rather than an outcome; Ng et al., 2016).

### Patient Perspectives

Recent literature also explores the concept of recovery from BPD from the perspective of the patient. A recent qualitative study explored the meaning of recovery with service-users who had been diagnosed with BPD, revealing themes in three main clusters: personal goals related to recovery, the balance between individual goals and treatment focus, and how recovered individuals feel (Katsakou et al., 2012). Personal goals for recovery identified by the participants were self-acceptance, building self-confidence, ability to control negative moods and cognition, improving relationships, practical achievements and employment, and reduction of suicidality, self-harm, and symptoms of other mental health issues such as substance use or post-traumatic stress. However, with respect to goals, about half of the sample noted that there was tension between their personal goals and the focus of treatment. Perspectives on recovery also varied widely, including participant statements indicating that they had made no progress, progress fluctuated, that they had improved but not fully recovered, or that they had recovered. Many participants (N = 24) struggled with the use of the word “recovery” or believed that full recovery would not be possible. Only five of the 48 service-users identified themselves as recovered, and of these, only one maintained this view throughout the interview.

Another qualitative study focused on the *process* of recovery among women with BPD who were in treatment (Holm & Severinsson, 2011). Thirteen women who were diagnosed with BPD and had previous suicidality were interviewed; the vast majority of whom (N=11) continued to struggle with suicidal behavior. The authors report two main themes, “struggling to assume responsibility for self and others” and “struggling to stay alive by enhancing self-development” (Holm & Severinsson, 2011, p. 168). The struggle to assume responsibility for self was described as being related to taking responsibility for one’s own life versus leaving the responsibility with someone else, being understood for who one is, and refusing to be violated (e.g., by one’s partner). The struggle to stay alive by enhancing self-development was characterized by various turning points in the participant’s attempts to navigate a life of unendurable emotional pain including working to feel safe and trusted. These authors conclude that “women with BPD who exhibit suicidal behavior can change when they feel confirmed, safe, and trusted” (Holm & Severinsson, 2011, p. 171).

Most recently, narrative inquiry has been applied to investigate the recovery experiences of women with BPD (Lariviere et al., 2015). Participants in this study had a formal diagnosis of BPD and must have participated in at least two years of a specialized treatment program for individuals with personality disorders. The authors assert that these criteria help to ensure that recovery was taking place in participants. However, it is unclear to what extent recovery was achieved by participants given that the specific nature of the treatment program and extent to which participants were actively engaged is not reported. Lariviere and colleagues (2015) used the person, environment, occupation model to organize dimensions of recovery from BPD. The majority of recovery dimensions were related to the individual, and these include constructs such as having hope, enjoying life again, cessation of suicidal thoughts, being more optimistic or realistic, and letting go of the past. With regard to the environment, participants emphasized the importance of having healthy relationships. In terms of occupational definitions, participants expressed ideas such as being able to care for oneself, being able to keep a job, having a meaningful role, and maintaining responsibilities. While participants often used different words to describe their experiences or perspectives of recovery from BPD, all seemed to convey a sense of focus on wellness and a process of working toward wellbeing and life satisfaction (Lariviere et al., 2015).

Although qualitative research with individuals with BPD is available, research that focuses on an evidence-based treatment for this population while using structured criteria to define recovery is not currently available. It is possible that one reason for this is that BPD has historically been viewed as a chronic and intractable condition. Although Zanarini and colleagues (2012) offer one definition of recovery, no qualitative researchers have used this as a guide in recruiting research subjects to investigate patient perspectives or experiences of recovery from BPD. The current study attempts to build upon previous findings by using more structured and detailed inclusion criteria in order to investigate the recovery experiences of individuals who have been diagnosed with BPD, and successfully treated with DBT. Furthering this area of research is needed because BPD is often viewed as a chronic condition. Given that some individuals do consider themselves to be recovered, it is important to better understand what recovery means. This is important for mental health professionals because it may help reduce stigma and bias against people with BPD that can interfere with treatment. However, it is also critically important to provide patients with BPD as well as their friends and family members with hope and concrete information about what may be expected in recovery following treatment with DBT.

## Reflexivity

We focused on awareness of our own expectations and biases throughout the design, data collection, and analysis phases of this project. The first author (CDC), who designed the parent study, is a postdoctoral scholar and professional counselor specializing in the treatment of BPD with DBT; the majority of her research is focused on applications of DBT with college students. The second author (ARS) is a master’s level social work student and research assistant, with cursory knowledge of DBT theory and practices. The differences in our clinical training and theoretical background presented us with both opportunities and challenges. While CDC was able to use the language of DBT during interviews and code transcripts for DBT-specific concepts (e.g., dialectical thinking), the great difference between us regarding knowledge of DBT and BPD required active discussion throughout the analysis process. However, we also believe it is important to view the data with beginner’s eyes (i.e., being open to whatever participants communicate without expectations). In this regard, ARS played an important role in the applications of codes and subsequent analysis. TRG joined the study during the data interpretation phase, in order to lend her expertise in practice and theory of DBT and knowledge of the extant literature in these areas. Throughout the analysis, we worked to create a balance between the influences of experience and openness.

## Method

### Participants

The participants in this study were five females and one male, aged 30–44. Four of the participants were in a romantic relationship and two were single. All participants had been previously diagnosed with BPD and treated with comprehensive DBT (i.e., the full DBT treatment package as described in Linehan, 1993). In order to be eligible to participate, all participants gave their consent for the first author to speak with their current or former DBT therapist. Therapists were required to confirm that they had at least intensive training (a 10-day training process) in DBT and that they had offered/were offering comprehensive DBT to the participant. Therapists also confirmed that participants had reached at least stage three of DBT. In other words, the therapists confirmed that participants had successfully overcome the challenges of significant behavioral (stage one) and emotional (stage two) dyscontrol and were now focused on dealing with ordinary problems in living (stage three). A detailed description of the recruitment procedures and further detail regarding the participant characteristics can be found in Chugani (2016). The Institutional Review Board at University of South Florida approved all study procedures and participants provided their written informed consent to participate. No compensation for participation was provided.

### Analysis

We conducted a thematic analysis using Braun and Clarke’s (2006) six-phase process. We chose these methods for two primary reasons: (1) Braun and Clarke’s method is well known and accepted in the field of psychological research, a primary audience for this work, and (2) Thematic analysis may be a relatively easier concept to understand (relative to phenomenology) for laypersons, such as individuals with BPD, who are a second primary audience for this work. Interviews were transcribed verbatim (with the exception of anonymization) by a professional transcription service and checked for accuracy by the first author. Prior to coding, two authors reviewed the transcripts in depth to familiarize themselves with the data. Given her knowledge of BPD and DBT, the first author generated the first draft of the codebook. All analysis procedures were conducted using Dedoose software, an online qualitative analysis tool that allows for multiple coders to work on a transcript in real-time. Initial codes were generated using inductive coding; codes were subsequently grouped into themes as the first author identified logical groupings. We next reviewed the codes together and co-coded sections of transcript until the second author felt confident in her knowledge of the codebook and the coding process. The second author then reviewed all of the coded transcripts, adding additional coded excerpts, re-coding excerpts where needed, and creating additional codes. Disagreements in code applications were discussed until a mutually agreeable resolution was reached.

Once we were satisfied that no more codes could be generated and all examples of each code had been identified, we reviewed and refined the themes. Themes were developed in an iterative fashion by: (1) grouping relevant codes into logical categories, (2) reviewing codes and themes for consistency throughout the transcripts, and (3) ongoing refinement of themes. In order to reach our final model, we established guidelines for retaining codes and themes for inclusion in the final analysis. Returning to our idea of achieving a balance in perspective, we decided to use two guidelines for retaining codes: (1) any code (regardless of level) appearing in at least 50% of the interviews would be retained, and (2) “outlier” codes that were not as frequently applied, but were clearly relevant to the research questions and did not significantly overlap with any other codes, would be retained. We began by creating a table of all of the codes and the frequency of their application. For any code applied in less than 50% of the interviews, we discussed the relative importance and uniqueness of the code until consensus was reached regarding whether it should be retained in our final analysis.

## Results

In total, we identified six themes, with level one and two codes associated with each. Table 1 provides an overview of the themes and their associated codes.

### Beliefs about recovery

Participants expressed varied viewpoints regarding their beliefs about recovery from BPD and whether full recovery was possible. While participants shared that they had made great progress toward recovery, they also questioned whether *full* recovery was possible. Some participants also expressed the belief that BPD would never fully remit. However, several participants also asserted that full recovery from BPD is possible. One outlier code that we found particularly useful in understanding one participant’s conceptualization was the idea that recovering from BPD is not like the process of recovering from alcoholism. One participant expressed this as follows, “I don’t really see where I am today in a state where someone who’s an addict is in recovery for the rest of their life, that they know that just a few drinks could put them over the edge. I don’t really feel like that edge is so close anymore. I feel like there’s maybe a mountain, but I’m going to know that I’m climbing it. I don’t feel like I’m going to get to the edge of that.” We interpret this as a dialectical view of recovery, unlike alcoholism, in which having a single drink could be viewed as relapsing (i.e., an all or nothing view of recovery). On one hand, there is a continuous process that they must engage in to stay recovered (knowing one is climbing a mountain). On the other hand, the process is not so unstable that even a minor slip could cause a swift decline back into mental illness (the edge is not so close anymore).

### Characteristics required for recovery

Characteristics required for recovery encompassed a broad set of ideas regarding factors that facilitate the recovery process. These included having awareness or realization that things needed to change, making changes to one’s environment in a manner that supported recovery (e.g., going to graduate school, moving away from one’s family, or generally seeking out people or environments that were more positive), and attending and engaging actively in therapy (coded as “doing the work”). All participants mentioned, and many emphasized, using their DBT skills. They also noted the importance of perseverance (not giving up on recovery even when it is very difficult), reducing impulsivity (having the ability to stop and think before acting or reacting), being able to accurately identify, label, and understand one’s emotions, and having willingness to change.

### Current experience of self

This theme refers to the manner in which the participants viewed themselves at present and their experiences as they went through their daily lives. Most participants expressed that they experienced continued symptoms of various types (e.g., struggling in social interactions, anxiety, depression, or thoughts of self-harm) as well as specific challenges with having heightened emotional sensitivity. As one participant describes it, “I care about other people so I kind of get hurt more, I’m always going to be sensitive, you know, I’m a highly sensitive person.” While some participants seemed to note the challenges of emotional sensitivity, others seemed to find positive attributes for this characteristic. Another participant explained, “It’s part of the liability of having all these feelings, but it also makes me great at what I do. So it’s a balance. It’s its own dialectic.”

In contrast, half of the participants also described themselves as normal, or more specifically, that their problems were normal problems that anyone might have, as opposed to problems that arose from BPD pathology. Two participants also described experiences related to stigmatization, which was sometimes internalized. As one participant explains, “What’s happened over the years is that people, even professionals, have labeled BPD and have said that you’ll never recover, you’ll have this for the rest of your life, until I met [my therapist]. But before that it was always like you’ll always be this way. You’re going to be difficult to treat…I’ve internalized a lot of that.”

### External supports to recovery

While the majority of the interviews were focused on the recovery experience and definition, the interviewer (CDC) also made a point of asking participants about what (if any) external elements were particular important in their recoveries. Participants’ responses included family members or partners and medication management, but the only code to meet our criteria for inclusion (i.e., present in 50% or more of the transcripts) in this theme was therapy and/or the individual therapist.

### Facets of recovery

This theme encompassed a wide variety of items that were expressed as pieces of being recovered, doing recovery, or descriptive of recovery. All of the participants mentioned being able to practice acceptance (e.g., of oneself and others) and having healthy relationships as part of their experience of recovery. Dialectical thinking (e.g., being able to see the truth in multiple, conflicting perspectives) was also emphasized by most of the participants. For example, one participant explained, “I used to think that people were all bad or all good, like, depending on what they had done. I don’t do that anymore. I’m able to see that people have flaws and they’re still good people.” Most participants also noted various elements of making and having meaning in their lives (defining one’s own life worth living, taking steps to make life meaningful, purposeful, and fulfilling), and some also specifically referred to contributing (e.g., being able to help others or contribute to society) as a primary source of meaning.

All participants also expressed the idea that they believed in their own abilities to manage or function (coded as “self-efficacy”). For many participants, having more positive emotional experiences was another primary characteristic of recovery, with happiness and peace being mentioned frequently. Having a connection with God or one’s personal sense of spirituality, as well as experiencing a reduction in the symptoms associated with BPD were also noted. With regard to BPD symptom reduction, there was a good deal of variation within this code. For example, one participant stated, “I think you can get an abatement from a lot of the symptoms, but I think a lot of your life is managing the fact that you don’t want to go back to where you were” while another stated, “I no longer meet the criteria [for BPD].” The majority of participants also noted that recovery is a process, which is in some cases, very difficult. One participant described the recovery process thusly, “I guess it’s a process. I guess that would be the best way to put it…I think it’s a daily activity to stay…recovered.” Another participant explained, “It was just a gradual process. So it was supported to happen that way and keep building on itself.” While participants had differing views of the recovery process, they generally agreed that it was a process that unfolded slowly.

### Motivating factors

Motivating factors were areas that participants described as helping to motivate them to continue on toward recovery. For some participants, motherhood and the desire to be there for their children was a powerful motivating factor. As one participant describes, “I feel that being a mom and taking care of my kids kept me going for much of the time.” Several participants also described various turning points; these tended to be powerful experiences that prompted change. For example, one participant explains, “I feel like it was like I have that first larger “aha moment” about DBT, where some of the skills come to be without me making an effort.” Another explained that after losing custody of her daughter she realized, “in order to get out of all of that trouble, I had to be compliant.”

## Discussion

When this project was originally designed, the primary aim was to uncover the *definition* of recovery from BPD. However, what we found was a complex and often contradictory set of ideas and experiences that seem to convey the *meaning* of recovery for individual participants. Many of the findings are dialectical in nature, containing seemingly conflicting concepts that stand side by side as truths. A dialectical perspective is often described as a “both, and” perspective (as opposed to the “either, or” perspective). With regard to research, although conflicting results are often difficult to interpret, in this case, the nature of the results further reinforces the clinical utility of a dialectical stance in the treatment of BPD: being able to tolerate or resolve dialectical tension is important for individuals with BPD because it may both contribute to recovery as well as continue to be a significant part of their experience in recovery.

One dialectic that was particularly striking was between the perception of recovery as possible and not possible. We assert that while individual beliefs about the possibility and permanency of recovery will differ depending on individual experience, it may also be that some participants question whether full recovery is possible because once an individual is labeled as mentally ill, it can be difficult to determine the source of subsequent emotional difficulties. While it is fairly straightforward to determine recovery from certain diseases or disorders (e.g., prolonged cancer remission is sometimes considered as full recovery) and there are objective measures of what is considered the disease versus health state in physical disease, measuring emotional health is much less clear.

Thus, it is possible that some of the conflict regarding whether recovery is possible may originate from participants’ beliefs about the difference between “normal” emotional distress and “pathological” emotional distress. For example, suppose a person who has recovered from BPD then experiences marital infidelity. This situation would likely result in feelings of abandonment, betrayal, intense anger, extreme emotional conflict, perhaps vacillating between loving and hating one’s partner. The reactions can be characteristic of BPD, and they may be considered normal reactions to infidelity. However, because there is no objective framework for measuring and categorizing emotional reactions, individuals in recovery from BPD (along with their families, friends, and providers) may wonder whether their distress is a “borderline” reaction.

The theme “current experience of self” revealed a similar dialectic: participants shared viewing themselves and their problems in living as normal (i.e., not different from what any other adult might experience) and, that they continued to have heightened emotional sensitivity. Those who described emotional sensitivity seemed to have accepted this as a part of themselves, with one participant offering a dialectical viewpoint (i.e., that it was both a challenge and a strength). These findings lend support for Linehan’s (1993) biosocial model of the development of BPD, which asserts that BPD is the product of transactions between biological vulnerabilities and an invalidating social environment. Both the biological and social components of this theory have empirical support. For example, abuse is considered to be a powerful form of invalidation. Individuals with BPD have been found to be significantly more likely to experience physical or emotional abuse by a caretaker and sexual abuse by a non-caretaker than individuals with other personality disorders; overall, 92% of individuals with BPD report being abused or neglected during childhood (Zanarini et al., 1997). Due to the high prevalence of severe invalidation in individuals with BPD, DBT treatment focuses on helping individuals with BPD get out of invalidating environments, receive treatment for the sequelae of severe invalidation (e.g., prolonged exposure for treatment of post-traumatic stress disorder), learn how to establish limits within environments to guard against future invalidation, and tolerate distress and regulate emotional reactions when invalidation or other emotional pain inevitably occurs.

Research using psychophysiological ambulatory assessment (repeated assessment of physiological and psychological indicators using technology that subjects can wear or carry with them as they go through their daily tasks) has also revealed that individuals with BPD self-report more frequent and intense emotions as well as more negative than positive emotions (Ebner-Priemer et al., 2007). Neuroimaging research has also revealed that treatment with DBT results in decreased activity in the amygdala (an area of the brain associated with emotional experiencing) as well as corresponding significant reductions in self-reported difficulties in regulating emotions (Goodman et al., 2014). The aforementioned evidence demonstrating the efficacy of DBT in addressing both biological and social elements is largely consistent with the experiences described by participants in this study.

One facet of recovery that may contribute more generally to the meaning of recovery is having healthy relationships, an area emphasized by all of the participants. In reviewing the 20 excerpts attached to this code, there is surprisingly little variability, with all participants conveying a deep sense of satisfaction at having achieved lasting and meaningful relationships. This finding is particularly interesting, considering recent research indicating that even among individuals who are in remission from BPD (defined as meeting less than three of the diagnostic criteria for BPD over the previous two years), there is a comparatively higher experience of rejection sensitivity (RS, an important construct related to interpersonal dysfunction; Bungert et al., 2015). Bungert and colleagues (2015) explain that individuals with BPD “need more security of acceptance to participate in social interactions, but the enhanced RS prohibits exactly this feeling of security about being accepted by others” (p. 9). Given that our findings suggest that individuals with BPD can achieve the security necessary to meaningfully engage in significant relationships, more research is needed to further elucidate changes in interpersonal sensitivity, security, engagement, and self-reported satisfaction throughout the treatment and recovery process.

A final facet of recovery described by most of the participants that may be a more general indicator of recovery is the experience of making and having meaning, through contributing to others or via other means. Although we defined recovery as reaching stage three of DBT (i.e., overcoming the behavioral and emotional dyscontrol associated with stages one and two in order to be able to focus primarily on ordinary problems in living), making and having meaning may be considered part of stage four of DBT, which is characterized by fulfillment and the capacity for freedom and joy (Behavioral Tech, LLC., n.d.). However, this emphasis on meaning and contributing is not altogether surprising, as DBT includes a substantial focus on identifying and working toward the individual’s goals that make life worth living. The act of contributing is also included as a skill for tolerating distress (Linehan, 2015).

In closing, the themes presented in this research are broad domains that reflect various aspects of the recovery process or experience. However, the extent to which each of these domains will contribute to individual meaning of recovery will vary. The individual’s perspective is also important with respect to enduring difficulties and the need to be mindful of these challenges. In other words, all individuals have vulnerabilities that differentially impact the potential consequences of our choices; the individual’s perception of his/her own vulnerabilities is critical. In this way, recovery from BPD includes not only more traditional markers of behavioral, emotional, and interpersonal health, but also a shift toward viewing oneself as an ordinary individual whose feelings and reactions are, for the most part, valid responses to one’s experiences. A key element in achieving this viewpoint is holding the belief that recovery is possible. Yet, that is not likely to occur for an individual receiving treatment from a clinician who does not hold this belief. Future research should investigate the relationship between beliefs about recovery, hope, and progress toward/maintenance of recovery in individuals with BPD. Finally, these findings advocate for continued efforts to destigmatize mental illness and BPD, as this will significantly diminish individuals’ suffering and maximize hope for recovery.

“Hope is like a road in the country; there was never a road, but when many people walk on it, the road comes into existence.”

– Lin Yutang

## Figures and Tables

**Table 1:** 

Themes	Level 1 Codes	Level 2 Codes

Beliefs About Recovery		

	BPD Persists	
	Not like an alcoholic^a^	
	Questioning full recovery	
	Recovery is possible	

Characteristics Required for Recovery		

	Awareness or realization	
	Changes environment	
	Doing the work	Using skills^b^
	Perseverance	
	Reducing unpulsivity	
	Understanding emotions	
	Willingness to change	

Current Experience of Self		

	Continued symptoms	
	Emotional sensitivity/challenges	
	Normal	
	Stigma or internalized stigma^a^	

External Supports to Recovery		

	Therapy/therapist	

Facets of Recovery		

	Acceptance^b^	
	Dialectical thinking	
	Healthy relationships^b^	
	Making and having meaning	Contributing
	Positive emotional experiences	Happiness
		Peace
	Recovery as a process	Difficult process
	Self-efficacy^b^	
	Stability/Consistency	
	Spirituality	
	Symptom reduction	

Motivating Factors		

	Motherhood/children	
	Turning point	

a Outlier codes (those appearing in less than 50% of the transcripts)

b Codes appearing in 100% of the transcripts
